# Galectin-1 promotes angiogenesis and chondrogenesis during antler regeneration

**DOI:** 10.1186/s11658-023-00456-7

**Published:** 2023-05-15

**Authors:** Xunsheng Li, Wanwan Shi, Guanning Wei, Jinpeng Lv, Datao Wang, Baorui Xing, Jue Zhou, Jianwei Zhao, Hongmei Sun

**Affiliations:** Institute of Special Economic Animal and Plant Sciences, Chinese Academy of Agricultural Sciences, Jilin, China

**Keywords:** Galectin-1, Antler stem cells, Regeneration, Crispr/cas9, Angiogenesis, Chondrogenesis

## Abstract

**Background:**

Deer antlers are the only known mammalian structure that undergoes full regeneration. In addition, it is peculiar because when growing, it contains vascularized cartilage. The differentiation of antler stem cells (ASCs) into chondrocytes while inducing endochondral extension of blood vessels is necessary to form antler vascularized cartilage. Therefore, antlers provide an unparalleled opportunity to investigate chondrogenesis, angiogenesis, and regenerative medicine. A study found that Galectin-1 (GAL-1), which can be used as a marker in some tumors, is highly expressed in ASCs. This intrigued us to investigate what role GAL-1 could play in antler regeneration.

**Methods:**

We measured the expression level of GAL-1 in antler tissues and cells by immunohistochemistry, WB and QPCR. We constructed antlerogenic periosteal cells (APCs, one cell type of ASCs) with the GAL-1 gene knocked out (APC^GAL-1−/−^) using CRISPR-CAS9 gene editing system. The effect of GAL-1 on angiogenesis was determined by stimulating human umbilical vein endothelial cells (HUVECs) using APC^GAL-1−/−^ conditioned medium or adding exogenous deer GAL-1 protein. The effect of APC^GAL-1−/−^ on chondrogenic differentiation was evaluated compared with the APCs under micro-mass culture. The gene expression pattern of APC^GAL-1−/−^ was analyzed by transcriptome sequencing.

**Results:**

Immunohistochemistry revealed that GAL-1 was widely expressed in the antlerogenic periosteum (AP), pedicle periosteum (PP) and antler growth center. Western blot and qRT-PCR analysis using deer cell lines further supports this result. The proliferation, migration, and tube formation assays of human umbilical vein endothelial cells (HUVECs) showed that the proangiogenic activity of APC^GAL-1−/−^ medium was significantly decreased (*P* < 0.05) compared with the APCs medium. The proangiogenic activity of deer GAL-1 protein was further confirmed by adding exogenous deer GAL-1 protein (*P* < 0.05). The chondrogenic differentiation ability of APC^GAL-1−/−^ was impeded under micro-mass culture. The terms of GO and KEGG enrichment of the differentially expressed genes (DEGs) of APC^GAL-1−/−^ showed that down-regulated expression of pathways associated with deer antler angiogenesis, osteogenesis and stem cell pluripotency, such as the PI3K-AKT signaling pathway, signaling pathways regulating pluripotency of stem cells and TGF-β signaling pathway.

**Conclusions:**

Deer GAL-1, has strong angiogenic activity, is widely and highly expressed in deer antler. The APCs can induce angiogenesis by secreting GAL-1. The knockout of GAL-1 gene of APCs damaged its ability to induce angiogenesis and differentiate into chondrocytes. This ability is crucial to the formation of deer antler vascularized cartilage. Moreover, Deer antlers offer a unique model to explore explore how angiogenesis at high levels of GAL-1 expression can be elegantly regulated without becoming cancerous.

**Graphical Abstract:**

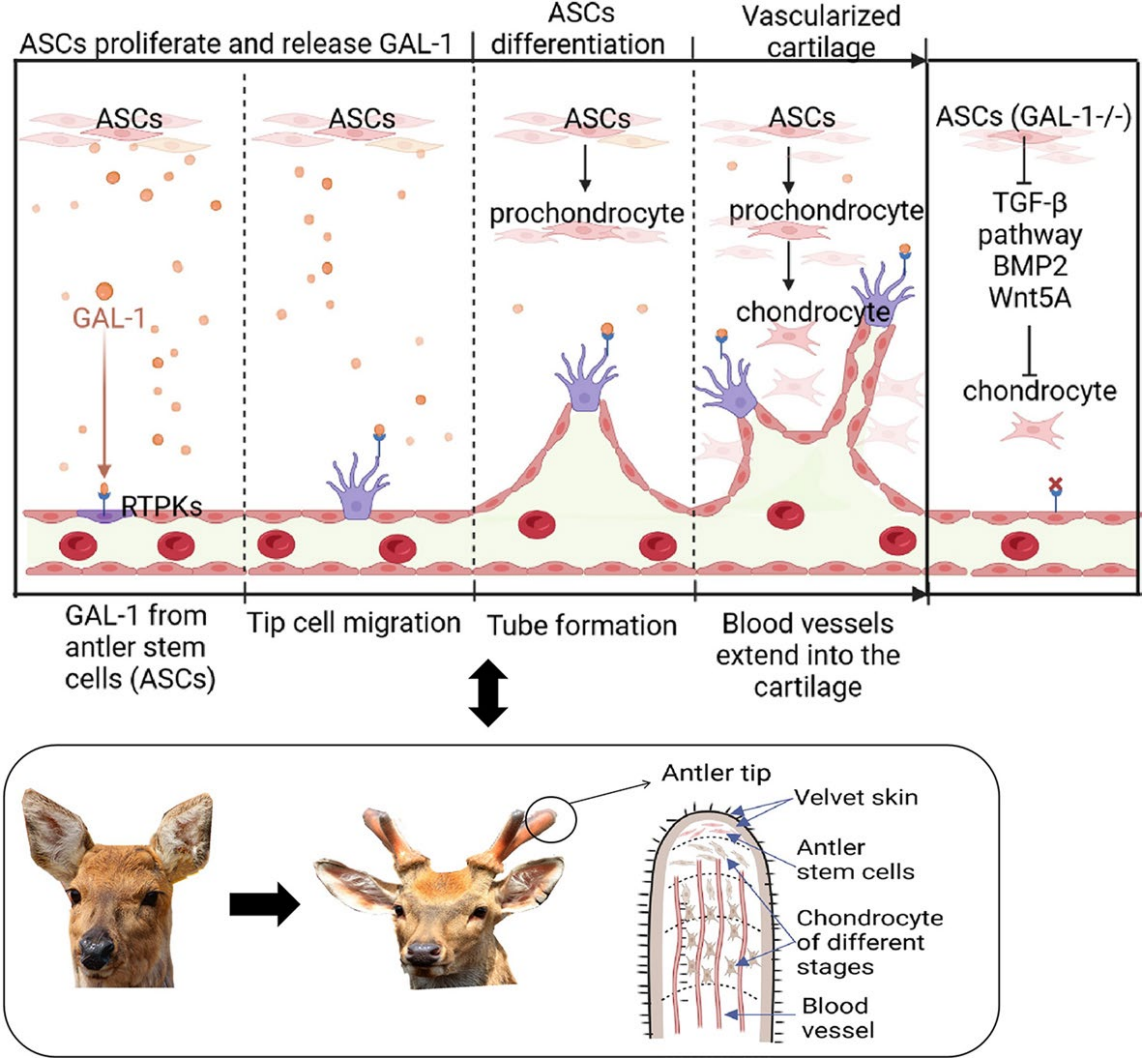

**Supplementary Information:**

The online version contains supplementary material available at 10.1186/s11658-023-00456-7.

## Background

Deer antler, a large mammalian bony structure, is annually cast and immediately after, it regenerates fully from the permanent bony protuberances known as pedicles [1, 2]. Hence, it offers the opportunity to explore how nature has solved the regeneration of a mammalian structure. Antlers mainly comprise cartilage, bone, skin, blood vessels, and nerves. Previous studies have demonstrated convincingly that annual antler renewal is a stem cell-based epimorphic process driven by cells resident in the pedicle periosteum (PP) [3]. The PP is the direct derivative of antlerogenic periosteum (AP), a tissue from which a deer pedicle and an initial antler develop [4]. The PP, AP and reserve mesenchyme (RM, for rapid antler growth) cells have stem cell attributes, so they are named antler stem cells (ASCs) [5]. In spring, the ASCs are activated, totally dead antlers are cast from their pedicles, and regeneration of new antlers immediately follows [6]. In late spring and early summer, antlers grow very fast (up to 2 cm/day) because of the rapid proliferation and differentiation of ASCs [7, 8]. Studies showed that the developed vascular system in antlers provides a nutritional basis for rapid growth and even forms unique vascularized cartilage that can self-repair and regenerate [9, 10]. Additional data revealed that the regeneration process and the rapid growth of antlers are achieved through mechanical stimulation and chemical induction of a number of molecules [11, 12]. However, which molecules are involved in regulating deer antler tissue regeneration, such as vascularized cartilage, remains to be determined. A better understanding of the mechanism of deer antler regeneration may contribute to the research in the field of regenerative medicine and may also open the way for innovative treatments of many diseases, such as cartilage repair, osteoporosis, and ischemic osteonecrosis.

One of the molecules involved in antler regeneration may be galectin. Galectins are a family of carbohydrate-binding proteins with an affinity for β-galactosides [13]. Galectins secreted to the outside of cells through atypical pathways have a wide role in regulating cell functions by binding to glycans in the cell membrane and extracellular matrix [14, 15]. Among these, Galectin-1 (GAL-1), a 135-amino acid protein, plays a critical role in embryonic development and angiogenesis. In GAL-1 deficient pregnant mice, fetal growth is delayed due to insufficient vascularization of the placenta [16]. In addition, GAL-1 expression was positively correlated with microvascular area in various types of cancer, such as hepatoma, gastric carcinoma, myeloma and high-grade serous carcinoma [17–20]. In a previous study, GAL-1 was detected to be highly expressed in antler stem cells (ASCs) by a gel-based proteomic technique [21]. However, it remains to be elucidated whether deer GAL-1 regulates angiogenesis and chondrogenesis during antler growth.

The overall aim was to understand the effects of GAL-1 in deer antler angiogenesis and chondrogenesis. Here, we aimed to analyze the differential expression of GAL-1 in AP, PP, ASCs, and antler growth center. By knocking out the GAL-1 gene of APCs (APC^GAL-1−/−^), we sought to reveal whether deer GAL-1 promotes angiogenesis and chondrogenesis. In addition, to further reveal the regulatory mechanism of GAL-1 in APCs, we set out to carry out a transcriptome analysis of APC^GAL-1−/−^.

## Materials and methods

### Ethics statement

All animal-related experiments in the present study were performed in accordance with the guidelines of the Animal Care and Use Committee of Institute of Special Economic Animal and Plant Sciences (Permit Number: ISAPASAEC-2022-60B).

### Tissue collection

The antlerogenic periosteum (AP) of 8-month-old male deer (n = 3) and pedicle periosteum (PP) of 3-year-old male deer (n = 3) were collected immediately after slaughtering. The facial periosteums (FP, as a control tissue) were collected immediately after removing AP or PP (Additional file 1: Fig. S1). According to the protocol described by Li and Suttie [22]. Briefly, the collection sites were sterilized using iodine and 75% alcohol after removing the hair. The skin is incised with a scalpel to expose the periosteum. The tissues were sampled and washed with phosphate buffer saline (PBS) for primary cell culture or fixed for histology. The distal 5 cm tips of the main beam of each antler, which contains the reserve mesenchyme (RM), pre-cartilage (PC), transition zone (TZ), and cartilage (C), were collected at their 30 days of growth (n = 3). The harvested antler tips were sectioned sagittally along the median plane for primary culture or histology.

### Cell culture

The primary cell culture for antlerogenic periosteal cells (APCs), pedicle periosteal cells (PPCs), facial periosteal cells (FPCs), and antler tip reserve mesenchymal cells (RMCs) was carried out as per Li [23]. Briefly, the sampled tissues were cut into small pieces smaller than 1 mm^2^ with a sterile scalpel and digested in Dulbecco’s Modified Eagle’s Medium (DMEM, Gibco, 11965092, USA) containing 150 U/ml collagenases at 37 °C. The digested complex was cultured in DMEM medium containing 10% fetal bovine serum (FBS; Gibco, 10100147C, USA), streptomycin (0.1 mg/ml), and penicillin (100 U/ml; Gibco, 15140163, USA) in a humidified atmosphere with 5% CO_2_ at 37 °C. The primary cells were trypsinized and transferred into T75 cell culture flasks when reaching sub-confluent. Half the amount of the subcultured cells were used for the following experiments when cell density reached 90% confluence, and the rest cells were cryopreserved in liquid nitrogen in the frozen medium containing 50% FBS, 40% DMEM, and 10% dimethyl sulfoxide (DMSO). Human vascular endothelial cells (HUVECs) were purchased from KeyGEN (KeyGEN, Nanking, China) and cultured in DMEM medium containing 10% FBS, streptomycin (0.1 mg/ml), and penicillin (100 U/ml) in a humidified atmosphere with 5% CO_2_ at 37 °C.

### Immunohistochemistry (IHC) and hematoxylin–eosin (HE) staining

As previously described, the tissue samples were fixed in 4% paraformaldehyde for histology [24]. The tissue samples were cut into 5 μm thick sections (Leica, RM2235, Germany). Histological sections were deparaffinized in xylene, rehydrated in graded ethanol, and treated with 0.01 Mol sodium citrate solution at 100 °C for 10 min for antigen retrieval. The endogenous peroxidase activity of histological sections was quenched with 0.3% H_2_O_2_. The sections were incubated with anti-GAL-1 antibody (1:500; Abcam, ab240111, England) or anti-rabbit IgG (isotype control, 1:500; Beyotime, A7016, China) overnight at 4 °C. After washing with PBS, the sections were incubated with the horseradish peroxidase (HRP)-conjugated secondary antibody for 30 min at 37 °C. Finally, the sections were stained with diaminobenzidine (Maixin, DAB-0031, China) and treated with hematoxylin. For HE staining, sections were stained with hematoxylin and blue back with ammonia after rehydration. As a counterstain, eosin was used for the staining of alkaline substances. Finally, sections were dehydrated and mounted with neutral resin. All sections were viewed using a Precipoint M8 scanning microscope.

### Immunofluorescent (IF) staining

Immunofluorescent staining was carried out as described elsewhere [25]. Briefly, Cells were seeded at a density of 20,000 cells/well in 24-well plates. After 24 h of incubation, cells were washed with PBS and fixed with 4% paraformaldehyde for 10 min. For intracellular proteins, cells were permeabilized for 5 min using PBS with 0.3% Triton X-100. After washing with PBS three times, blocking was performed for 30 min using PBS with 3% Bovine Serum Albumin (BSA; Sigma, A1933BSA, Germany). The primary antibodies, including anti-GAL-1 (1: 500; Abcam, ab240111, England) and anti-rabbit IgG (1:500; negative control, Beyotime, A7016, China), were left overnight at 4 °C. The next day, the primary antibodies were washed off using PBS. The secondary antibodies, Alexafluor 594 goat antirabbit (Invitrogen, A-214291, USA) or Alexa fluor 488 (Invitrogen, A-11008, USA), were applied for 1 h (h) at room temperature (RT) in the dark. The nuclei of cells were counterstained with 2-(4-amidinophenyl)-6-indolecarbamidine dihydrochloride (DAPI) for 10 min at RT. Cells are covered with anti-fade reagent and examined under a fluorescent microscope.

### Western blotting

Total proteins were extracted from the cultured cells using RIPA lysis buffer (Beyotime, P0013, China), separated using 12% SDS-PAGE gel and transferred to PVDF membranes (Millipore, ISEQ85R, USA). The membranes were incubated with 5% nonfat dry milk for 2 h at RT and then overnight at 4 °C in PBS-diluted primary antibodies (1: 1000; Abcam, ab240111, England). The next day, after the membranes were washed three times with TBST, incubated with HRP-conjugated secondary antibody (1:1000; Beyotime, AF5003, China) for 1 h at RT. The levels of proteins were visualized using an ECL system (Tanon5800, China). The target protein bands were quantified by scanning densitometry using image j (v.1.6.0). GraphPad Prism (v. 8.0.1) was used to analyze data. The *t*-test was employed to calculate the differences between samples, and *P*-value < 0.05 was considered statistically significant (Additional file 2).

### Quantitative real-time PCR (qRT-PCR) analyses

Total RNA was extracted from the cultured cells using TRIZOL Reagent (Invitrogen, 15596018, USA) following the manufacturer’s protocol. First-strand cDNA was synthesized from 1 μg of total RNA (DNase treated) using a Primescript RT-PCR kit (Takara, RR047A, China). The specifific primers were designed using software Primer 5 (Additional file 3: Table S1). Real-time PCR was performed using SYBR green I Master mix (Takara, RR820A, Japan) on Roche Light Cycler 480 Real-Time PCR System. GAPDH was used in each reaction as a baseline control. PCR reaction conditions were set for 30 s at 95 °C, followed by 40 amplification cycles (95 °C for 10 s, 10 s at specific primer annealing temperature, and 72 °C for 30 s). The relative mRNA expression was calculated using the 2^−∆∆CT^ algorithm.

### Enzyme-linked immunosorbent assay (ELISA)

Adherent cells (1 × 10^6^) were washed with PBS and cultured with serum-free DMEM for 24 h. The supernatant was collected and used for ELISA. Follow the kit instructions (Cloud-Clone Corp, SEA321Bo, China). Briefly, 100 µl of sample and standard were added to a 96-well plate coated with GAL-1 antibody and incubated for 1 h at 37 °C. After washing with the cleaning buffer, prepared detection reagents were added sequentially. Finally, TMB chromogenic solution was used for color development in the dark and then read at 450 nm immediately. Protein concentration was calculated according to the standard curve.

### Cloning and hybridization of gRNA oligonucleotides

CRISPR—GuideRNA (gRNA) sequences were designed by CRISPR Online design website (https://zlab.bio/guide-design-resources; Additional file 3: Table S2). The three pairs of gRNAs were cloned to lentiGuide-Puro (Addgene, #52963) according to a modified protocol from Sanjana [26]. The LentiGuide-Puro was linearized with the Esp3I enzyme (ThermoFisher, ER0451, USA) according to the manufacturer's specifications. The linearized LentiGuide-Puro was separated by agarose gel electrophoresis and recovered by DNA purification kit (TIANGEN, DP209-02, China). The ligation reaction of the annealed gRNAs to the LentiGuide-Puro were set up using Quick Ligation Kit (NEB, M2200S, USA) and incubated at RT for 10 min. The recombinant LentiGuide-Puros were transformed into Stbl3 chemically competent bacteria (TransGen, CD521, China) following the manufacturer's protocol and verified ligation by Sanger sequencing (Completed by Shanghai Sangon Biotechnology Co., Ltd.).

### Virus production and establishment of knockout cell line

LentiCAS9-Blast (10 μg, Addgene, #52962) and recombinant LentiGuide-Puros (10 μg) were mixed with psPAX2 (8 μg, Addgene plasmid #12260) and pMD2.G (4.5 μg, Addgene plasmid #12259), respectively. The mixtures were transfected to 80% confluent 293T cells in the T175 flask using transfection reagents (Roche, 6365787001, Switzerland) according to the manufacturer's protocol. The viral supernatant was collected after 24, 48, and 72 h, filtered by a 0.45 μm filter (Millipore, SLHV033NS, USA), ultracentrifuged at 72,000×*g* for 2 h (BECKMAN, Optima™ XE-100, USA). For infection, 2 × 10^4^ APCs were infected with viral particles expressing CAS9 and gRNAs in the presence of 5 μg/ml polybrene (Beyotime, C0351, China). After 6 h, viral particles were replaced with fresh medium. After 72 h of infection, 4 μg/ml puromycin (Beyotime, ST551, China) and 5 μg/ml blasticidin (Beyotime, ST018, China) were added to the culture medium for double-drug screening until all cells in the control group died. All manipulations for monoclonal cells were performed under a microscope on a clean bench. A single suspended APC was inoculated in a 96-well plate by micropipette and expanded to obtain individual clone.

### T7 endonuclease I (T7EI) cleavage assay and Sanger sequencing verification

For clones’ validation, genomic DNA were extracted using the DNA extraction kit (TransGen, EE181, China). The PCR amplification was performed using High-Fidelity DNA Polymerase (Takara, R045A, Japan) with primers on-target (gRNA target site). The PCR products of the clones infected with lentivirus were mixed with the PCR product of wild-type cells in equal amounts. The mixtures were denatured and annealed to form heteroduplexes, followed by enzymatic digestion with 1 µl of T7EI (NEB, M0302S, USA) at 37 °C for 30 min. The products were resolved electrophoretically on a 1.5% agarose gel to analyze the DNA digestions. PCR products of T7EI-positive clones were inserted into the pEASY vector (TransGen, CB101, China) to identify the mutant alleles. Sanger sequencing was performed by Shanghai Sangon Biotechnology.

### Cell counting kit-8 (CCK8)

The relative proliferation rates of human umbilical vein endothelial cells (HUVECs) were measured using Cell Counting Kit-8 (Beyotime, C0041, China). In brief, cells were collected from the logarithmic growth phase and seeded into 96-well plates at a density of 2000 cells/well. At low serum concentrations (2%), HUVECs were cultured in DMEM with recombinant deer GAL-1 (20 ng/ml), which purified by Wang [27], DMEM with recombinant human VEGF (20 ng/ml, Peprotech, 100-20, USA; A growth factor that promotes the proliferation and migration of vascular endothelial cells, commonly used to promote angiogenesis) and conditioned medium of APCs, respectively. At 24, 48, 72, and 96 h, cells were incubated with 10 μl of CCK8 per well for 2 h (Beyotime, C0041, China). The absorbance was measured using a microplate reader at a wavelength of 450 nm, and the culture medium without cells was corrected and calculated as a control. The two-way ANOVA test was used to calculate the differences between samples, and *P-*value < 0.05 was considered statistically significant. GraphPad Prism (v. 8.0.1) was used to analyze data.

### 5-Ethynyl-2′-deoxyuridine (Edu)

The proliferation rates of HUVECs were measured using an Edu kit (Epizyme, CX002, China) according to the kit instructions. Cells were collected from the logarithmic growth phase and seeded into 24-well plates at a density of 1 × 10^4^. At low serum concentrations (2%), HUVECs were cultured in DMEM with recombinant deer GAL-1 (20 ng/ml), DMEM with recombinant human VEGF (20 ng/ml), and conditioned medium of APCs, respectively. After the cells were seeded for 48 h, add the same amount of 2×Edu working solution as the medium and incubate for 4 h. The cells were fixed with 4% paraformaldehyde and then incubated with 0.3%Triton X-100 for ten minutes at RT. Click reaction solution was used for nucleic acid staining of dividing cells. The nuclei were stained by Hoechst. Cells were recorded under a fluorescence microscope, and the proportion of proliferating cells was counted using image j (v.1.6.0). GraphPad Prism (v. 8.0.1) was used to analyze data. The t-test was employed to calculate the differences between samples, and *P-*value < 0.05 was considered statistically significant.

### Tube formation assay

The effects of deer GAL-1 on angiogenesis were measured using an endothelial cell tube formation assay as previously described by DeCicco-Skinner [28]. In brief, growth factor-reduced matrigel (Corning, 356231, USA) was diluted 1:3 with DMEM. Diluted matrigel (10 μl) was added to angiogenesis slides (Ibidi, 81531, Germany) and incubated at 37 °C for 45 min. In serum-free condition, 1 × 10^4^ cells/well were seeded slides and cultured in DMEM with recombinant deer GAL-1 (20 ng/ml), DMEM with recombinant human VEGF (20 ng/ml, as a positive control), and serum-free conditioned medium of APCs for 6 h at 37 °C with 5% CO_2_, respectively. Tube formation images were acquired by a microscope. The tube formation effects were analyzed by the angiogenesis analysis plug-in of image j 1.6.0. GraphPad Prism (v. 8.0.1) was used to analyze data. The *t*-test calculated the differences between samples, and *P-*value < 0.05 was considered statistically significant.

### Migration assay

The wound healing culture insert (IBIDI, 80209, Germany) was used for the wound healing assay. Each reservoir (0.22 cm^2^) was loaded with 70 μl medium containing 2.1 × 10^4^ HUVECs. The HUVECs were incubated in a medium supplemented with 10% FBS for 12 h at 37 °C under 5% CO_2_. After the cells reached confluence (12 h), the inserts and previous culture medium were removed, and then wells were filled with 400 μl/well of medium (DMEM with recombinant deer GAL-1 (20 ng/ml), DMEM with recombinant human VEGF (20 ng/ml, as a positive control) and serum-free conditioned medium of APCs) for 24 h at 37 °C under 5% CO_2_, respectively. After 24 h of incubation, the images were acquired by the microscope. The rates of scratch healing were analyzed by the plug-in of image j 1.6.0. GraphPad Prism (v. 8.0.1) was used to analyze data. The t-test was employed to calculate the differences between samples, and *P*-value < 0.05 was considered statistically significant.

### Micro-mass culture

The micro-mass culture was carried out following the method reported elsewhere [29]. Briefly, the APCs were cultured in T75 flasks, trypsinized when reaching 90% confluence, and resuspended in a chondrogenic medium (DMEM, 10 ng/ml recombinant human TGF-β1 protein (Peprotech, 100-21, USA), 50 μg/ml ascorbate-2-phosphate, 0.1 μM dexamethasone) to a concentration at 1 × 10^8^ cells/ml. 100 μl of cell suspension were seeded in the center of each well of 6-well plates and incubated for 3 h at 37 °C to facilitate adherence of the cells. Afterward, a chondrogenic medium (2 ml) was added to each well around the forming cell aggregate. The medium was subsequently replaced every 2 days. The cell nodules were harvested 3 weeks after initial seeding. After that, nodules formed from micro-mass culture were fixed in 4% formaldehyde, embedded in paraffin, and cut into 5 µm sections. For histological evaluation, the sections were stained with Alcian blue, Collagen II, and GAL-1.

### RNA-Seq

Total RNAs were extracted from the APC^WT^, APC^Vector^, and APC^GAL-1−/−^ using a Trizol reagent (Invitrogen, 15596018, USA) according to the manufacturer’s procedure. The RNA quality was confirmed through Agilent 2100, and 5 µg per RNA sample was used to construct the library. The tagged cDNA libraries were loaded onto the BGIseq500 platform (BGI-Shenzhen, China) and sequenced for single-end 50 bases read. The reads that contained the sequencing adaptor and unknown bases (> 5% “N” s per read) and the low-quality reads (> 15% bases smaller than Q20 per read) were discarded. The selected clean reads from each sample were aligned and mapped to the sika deer (*Cervus Nippon*) reference genome using Bowtie (v2.2.5, http://bowtie-bio.sourceforge.net/Bowtie2/index.shtml). The gene expression level and FPKM were calculated with RSEM. (v1.2.12, https://github.com/deweylab/RSEM). The gene expression patterns of APC^WT^, APC^Vector^, and APC^GAL-1−/−^ were compared through Principal Component Analysis (PCA) analysis and Pearson correlation coefficient, performed through the cor and princomp functions in R, respectively. The differentially expressed genes (DEGs) were identified using the DESeq2 R package (v1.4.5, http://www.bioconductor.org/packages/release/bioc/html/ DESeq2.html; visualized on the Tutools platform (https://www.cloudtutu.com) [30]. The adjusted *P*-value < 0.01 and |log_2_foldchange| > 1 were set as the threshold for significantly differential expression. The Venn analysis was performed to calculate the numbers of DEGs among APC^WT^, APC^Vector^ and APC^GAL-1−/−^ using Excel. The GO enrichment analysis and the KEGG pathway enrichment analysis on the selected DEGs were performed using KEGG Orthology Based Annotation System (KOBAS, http://kobas.cbi.pku.edu.cn) and visualized using the ggplot2 R package. The String platform (https://cn.string-db.org/) was used to perform the gene network analysis (*P*-value ≤ 0.01), and then major interaction networks were clustered by Cytoscape (v3.8.0, https://cytoscape.org/) [31].

### Statistical analysis

The numeric data are expressed as the mean ± SD. The t-test or two-way ANOVA test was used to test the statistical significance. *P-*value < 0.05 was considered statistically significant. GraphPad Prism (v8.0.1, www.graphpad.com) was used to analyze data.

## Results

### Expression of GAL-1 in AP, PP, antler growth center, and cultured ASCs

**Fig. 1:**
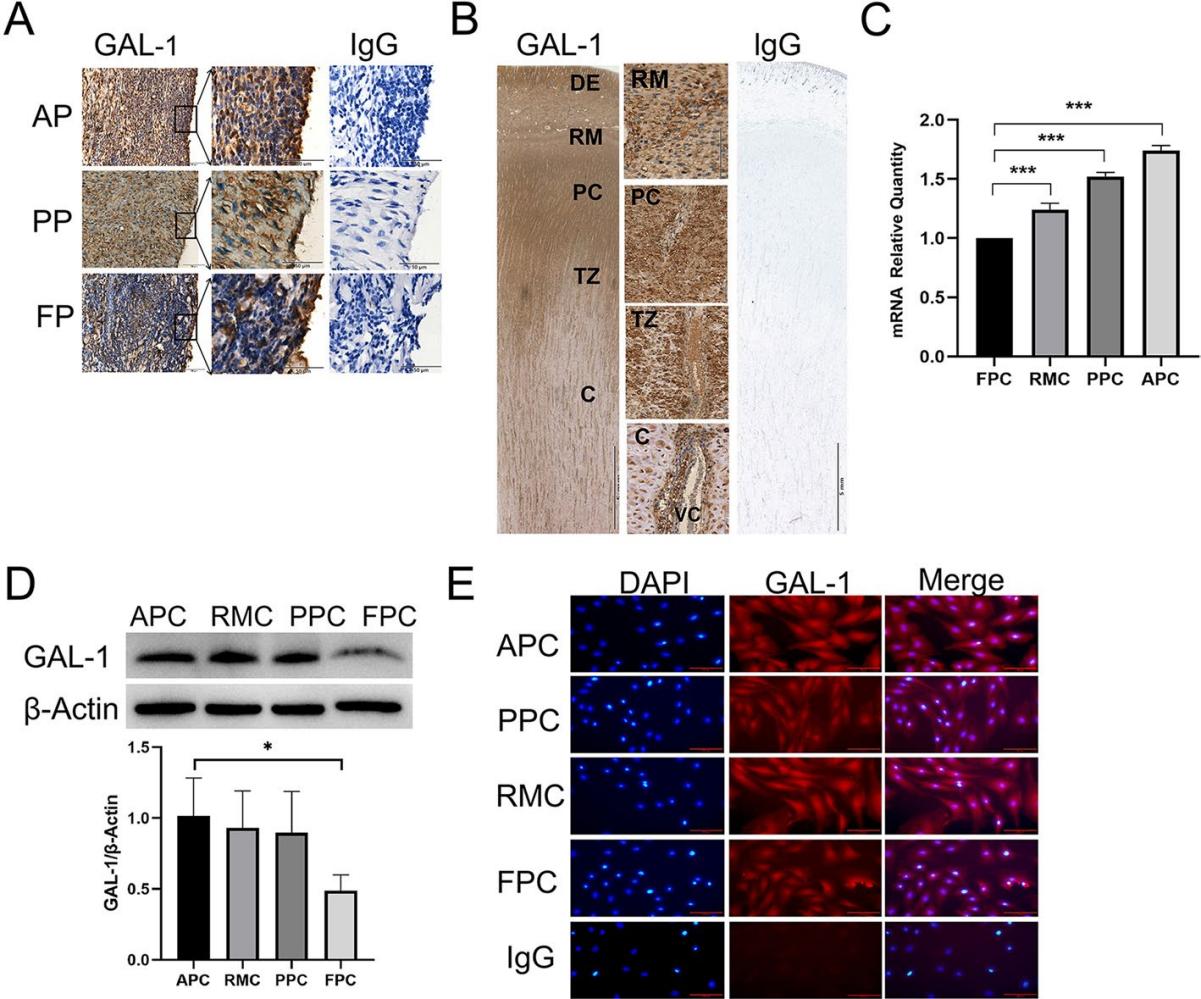
Immunohistochemical localization and expression of GAL-1 in the growth center of antler tip, AP, PP, and cultured ASCs. **A** Immunohistochemistry of GAL-1 of AP, PP, and FP (as a control), respectively. Magnification shows an enlarged view of the GAL-1, scale bar = 50 μm for GAL-1 and IgG. **B** Immunohistochemistry staining of GAL-1 in the antler tip, Scale bar = 5 mm for GAL-1 and IgG. Magnification shows an enlarged view of the GAL-1, scale bar = 100 μm. IgG shows rabbit IgG control. **C** The relative mRNA levels of GAL-1 in ASCs and FPCs (as a control) were detected by qRT-PCR. **D** Protein expression levels of GAL-1 in ASCs and FPCs (as a control) were detected using Western blotting. **E** Immunofluorescence staining of GAL-1 in ASCs and FPCs, scale bar = 100 μm. *DE* dermis, *RM* reserve mesenchyme, *PC* pre-cartilage layer, *TZ* transition zone, *C* cartilage, *AP* antlerogenic periosteum, *PP* pedicle periosteum, *FP* facial periosteum, *APCs* antlerogenic periosteal cells, *PPCs* pedicle periosteal cells, *RMCs* reserve mesenchymal cells, *FPCs* facial periosteal cells, *VC* vascular channel. Data are presented as the mean ± SD of three experiments, t-test, *P < 0.05, **P < 0.01, ***P < 0.001

In order to identify GAL-1 expressed tissues, we detected GAL-1 protein in AP, PP, and antler tip growth centers by immunohistochemistry. The majority of the cells (91 ± 7%) were specifically stained in AP tissues, to a to a lesser extent in the PP (87 ± 8%) and the least in the FP (45 ± 7%) (Fig. 1A). The cells were extensively stained from the distal to the proximal (RM, PC, TZ, to C) of the antler growth center (Fig. 1B). As mature chondrocytes were generated and cell density decreased, the staining of GAL-1-positive cells gradually became lighter, but cells associated with vascular channels (VC) remained heavily stained (Fig. 1B). These results demonstrated that GAL-1 was highly expressed in ASCs and its progeny, and persistently expressed in the vascular wall of blood vessels in the antler.

The expression levels of GAL-1 mRNA in the ASCs were investigated using qRT-PCR. All types of ASCs (APCs, PPCs, and RMCs) expressed high levels of GAL-1 mRNA compared with the FPCs (*P* < 0.001; Fig. 1C). The GAL-1 protein were highly expressed in APCs compared with FPCs (*P* < 0.05; Fig. 1D), which was also consistent with our immunohistochemical findings. Immunofluorescent results showed that the GAL-1 protein was localized in the nucleus and cytoplasm of antler cell lines (Fig. 1E).

### The GAL-1 gene of APCs was knocked out by CRISPR/CAS9

**Fig. 2:**
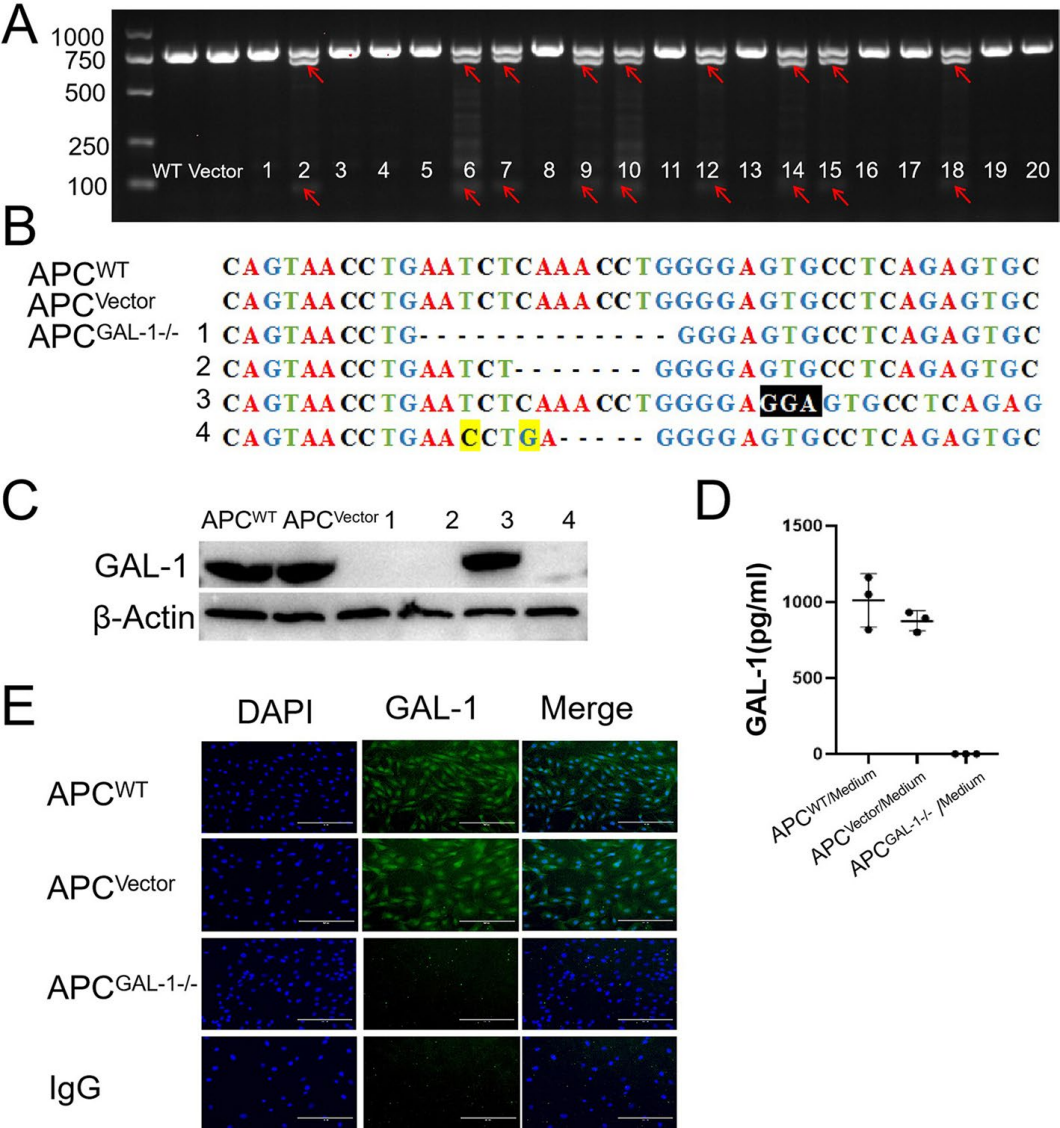
The construction of APCs with GAL-1 gene knocked out. **A** T7EI digestion products from twenty single-cell colonies were analyzed by agarose gel electrophoresis. DNA fragments of the GAL-1 gene with mutated sites were indicated by red arrows (2, 6, 7, 9, 10, 12, 14, 15, 18). **B** Sequencing of APC colonies with GAL-1 gene mutation. There were four types of mutations: 1. 13 bp deletion; 2. 7 bp deletion; 3. Insert 3 bp, black shading as substitution base; 4. 5 bp deletion and 2 bp replacement, yellow shading as substitution base; **C** the expression of GAL-1 protein in four mutant cell colonies were detected by Western blotting. The GAL-1 proteins of the 1st, 2nd, and 4th mutant cell colonies were completely knocked out. **D** Detection of GAL-1 protein in the serum-free culture medium of cell colonies by ELISA. **E** The expression of GAL-1 protein in cell colonies was detected by immunofluorescence, scale bar = 200 μm. APC^WT^: Wild-Type APCs; APC^Vector^: APCs infected with empty vector (no gRNA sequence attached); APC^GAL−1−/−^: APCs with GAL-1 gene knocked out

T7EI enzyme identification showed 9 mutant cell clones among the 20 cell clones (Fig. 2A). Four types of mutant sequences were confirmed in 9 mutant cell clones by sequencing (Fig. 2B). The results of Western Blot (Fig. 2C) and immunofluorescence (Fig. 2E) showed that GAL-1 protein was completely knocked out in the three mutant types of cell clones. GAL-1 is a secreted protein. We also detected the content of GAL-1 protein by ELISA in the culture medium 24 h after the cells were cultured and confirmed that there was no GAL-1 protein (Fig. 2D). The APC line with GAL-1 gene knockout (APC^GAL-1−/−^) were constructed successfully.

### The ability of APCs to induce proliferation, tube formation, and migration of HUVECs via paracrine was impaired due to the knockout of the GAL-1 gene

**Fig. 3:**
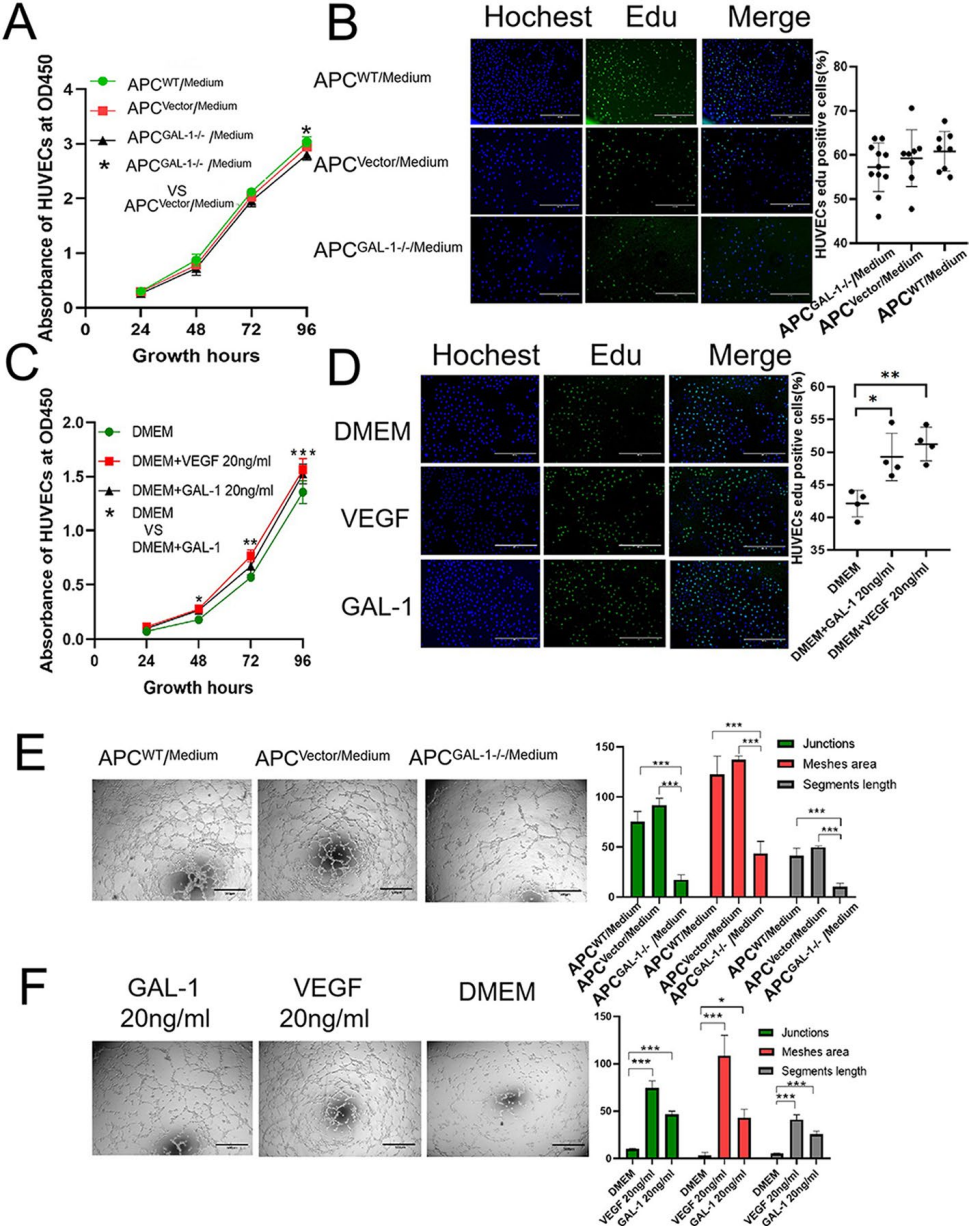

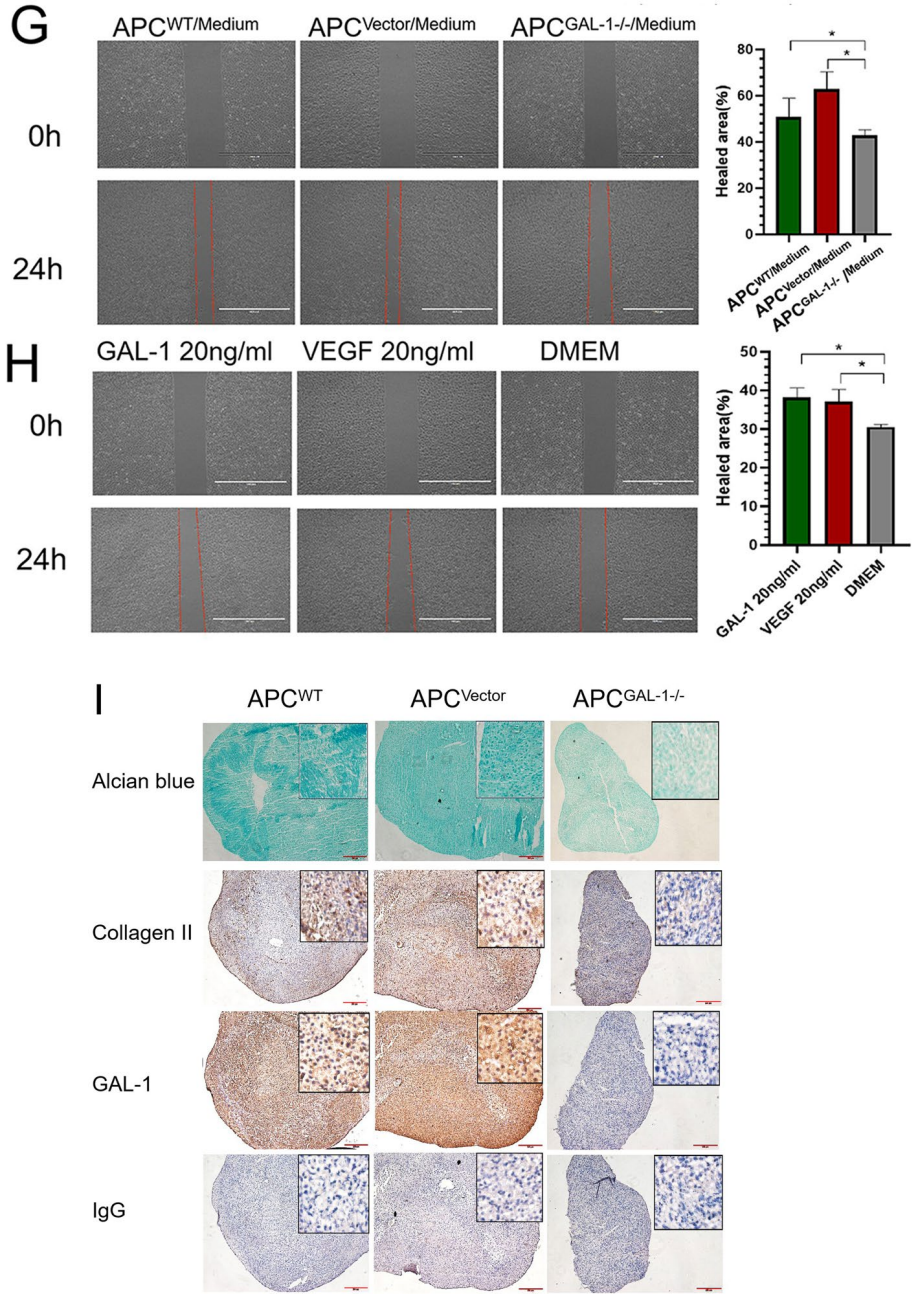
Effects of deer GAL-1 on angiogenesis and chondrogenesis. **A**, **B** The proliferation of HUVECs exposed to APCs-cultured medium was measured by Cell CCK8 (**A**; 24, 48, 72, and 96 h) and Edu assay (**B**; 48 h), respectively. **C**, **D** The proliferation of HUVECs exposed to exogenous deer GAL-1 (20 ng/ml) was measured by CCK8 (**C**; 24, 48, 72, and 96 h) and Edu assay (**D**; 48 h), respectively, as compared with control wells without deer GAL-1. **E**, **F** Tube formation assay of HUVECs exposed to APCs-cultured medium (**E**) and deer GAL-1 recombinant protein (**F**), respectively. **G**, **H** Migration assay of HUVECs exposed to APCs-cultured medium (**G**) and deer GAL-1 recombinant protein (**H**), respectively. **I** Effects of GAL-1 gene knocked out on chondrogenic differentiation of APCs. Chondrogenic differentiation of APCs grown as micro-mass cultures. Chondrogenic pellets were cut into 5 μm sections for Alcian blue staining and immunohistochemical staining for type II collagen and GAL-1. The Alcian Blue and Collagen type II protein staining depth of the resultant nodules were decreased in the APC^GAL-1−/−^ compared with the control. The results showed that APC^GAL-1−/−^ did not form cartilaginous nodules compared to the control group. Data are presented as the mean ± SD, t-test or two-way ANOVA test (CCK8 assay), *P < 0.05, **P < 0.01, ***P < 0.001

The HUVECs were cultured in a conditioned medium of APCs to detect whether APCs could induce proliferation, tube formation, and migration of endothelial cells by secreting GAL-1 protein. The results of the CCK8 and Edu assays showed that that there was no significant difference in the proliferation rate of HUVECs at 48 h (Fig. 3A, B). At 96 h, CCK8 showed that the proliferation rate of HUVECs cultured with APC^GAL-1−/−^ conditioned medium was significantly lower than that of the control group (*P* < 0.05; Fig. 3A). We believe that APC^GAL-1−/−^ conditioned medium still contains growth factors other than GAL-1, so a longer culture period is needed to reflect the effect of GAL-1 deficiency on the proliferation of HUVECs. The tube formation of HUVECs cultured in serum-free medium of APC^GAL-1−/−^ was significantly decreased compared with the controls. The quantification criteria include junctions (*P* < 0.001; Fig. 3E), meshes area (*P* < 0.001; Fig. 3E), and segments length (*P* < 0.001; Fig. 3E). Likewise, the rate of migration of HUVECs cultured in serum-free medium of APC^GAL-1−/−^ was decreased significantly compared with the controls (*P* < 0.05; Fig. 3G). Altogether, APCs can promote endothelial cell proliferation, tube formation, and migration by secreting GAL-1 protein.

### Angiogenic activity of deer GAL-1 protein in vitro

The role of deer GAL-1 protein in regulating the angiogenesis of endothelial cells was determined using cell proliferation, tube formation, and cell migration assays. The proliferation of HUVECs was detected by CCK8 (Fig. 3C) and Edu (Fig. 3D) assay. Our results showed that both 20 ng/ml GAL-1 and 20 ng/ml VEGF significantly increased the proliferation rate of HUVECs (*P* < 0.05), demonstrating a similar effect as shown by GAL-1 protein from VEGF. The tube formation assay showed that deer GAL-1 protein (20 ng/ml) significantly stimulated the tube formation of HUVECs compared with the control. The quantification criteria include meshes area (*P* < 0.05; Fig. 3F), segments length (*P* < 0.001; Fig. 3F), and the number of junctions (*P* < 0.001; Fig. 3F). Deer GAL-1 protein also promoted migration of HUVECs (*P* < 0.05; Fig. 3H) compared with the control. Likewise, treatment with VEGF also significantly increased the number of tube formation, and migration of HUVECs. Overall, deer GAL-1 protein has a strong angiogenic activity.

### Effects of GAL-1 gene knockout on the chondrogenic differentiation of the APCs in micro-mass culture

We performed micro-mass culture to evaluate the effects of GAL-1 on the chondrogenesis of APCs. The cartilage nodules formed by APCs in micro-mass culture were sectioned and stained. Notably, the cartilage nodules formed by APC^GAL-1−/−^ were smaller than those in the control group, indicating that it could not be induced into normal size cartilage nodules. This phenomenon may be related to the decrease of cell adhesion and the obstruction of differentiation process caused by the deletion of GAL-1 gene. The extracellular matrix from the APC^WT^ and APC^Vector^ nodules were heavily stained with alcian blue (Fig. 3I), whereas that from the APC^GAL-1−/−^ nodules were negative to the staining (Fig. 3I), suggesting that the APC^GAL-1−/−^ nodules did not contain sulfated proteoglycans, an essential component of cartilage matrix. Results of immunohistochemistry showed that the deficiency of the GAL-1 gene had suppressed the gene expression of type II collagen (a marker of chondrogenesis) in the nodules formed by the micro-mass cultured APC^GAL-1−/−^ compared to the control (Fig. 3I). Immunohistochemical staining of GAL-1 further demonstrated that there was no GAL-1 in APC^GAL-1−/−^ during micro-mass culture (Fig. 3I). These results indicated that chondrogenesis had taken place in the APCs nodules in the control groups, while knocking out the GAL-1 gene had impeded the progression of chondrogenic processes.

### Transcriptional analysis showed an altered gene expression pattern in APC^GAL-1−/−^

Comparative transcriptional analyses among APC^GAL-1−/−^, APC^WT^, and APC^Vector^ were performed to obtain a global view of the role of the GAL-1 gene in APCs. After filtration by quality control, a total of 387.08 million (58.05 Gb) bases were generated on the Illumina HiSeq sequencing platform. The Q30 base percentage of APC^WT^, APC^Vector^ and APC^GAL-1−/−^ data were 93.05%, 93.38% and 93.42%, respectively (Additional file 3: Table S3). The average percentage of valid data successfully annotated was 89.57% (Additional file 3: Table S4).

**Fig. 4:**
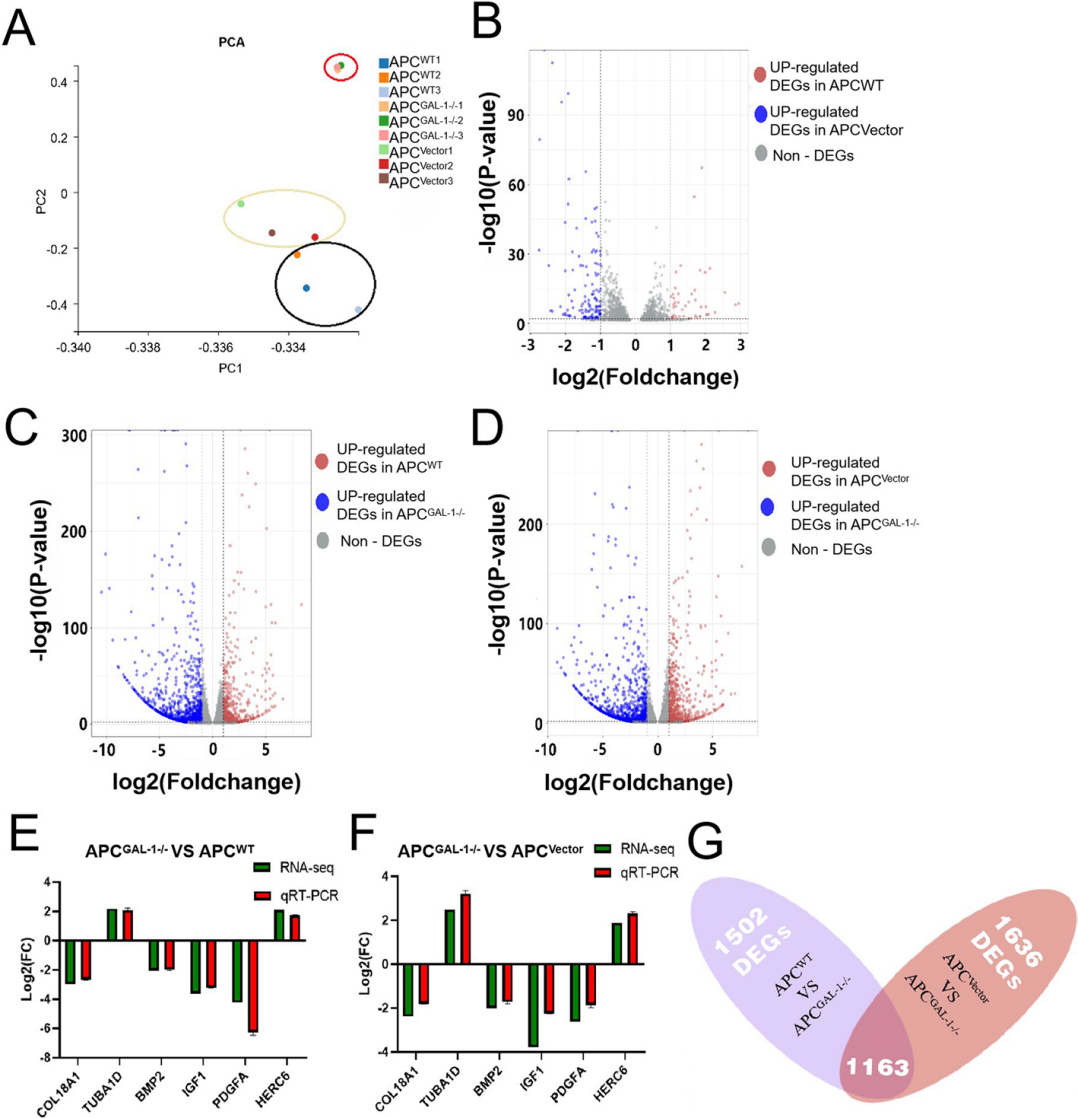
A general view of gene expression patterns and differentially expressed genes (DEGs) analysis on APC^WT^, APC^Vector^, and APC^GAL-1−/−^. **A** The PCA analysis was performed on the transcript signatures of APC^WT^, APC^Vector^, and APC^GAL-1−/−^. **B**–**D** The volcano plots showed the expression levels of DEGs between APC^WT^ and APC^Vector^ (**B**), between APC^GAL-1−/−^ and APC^WT^ (**C**), and between APC^GAL-1−/−^ and APC^Vector^ (**D**), respectively. Y-axis denotes the -log10 value of the adjusted *P-*value (padj) of DEGs, padj < 0.01. The X-axis represents the log2foldchange values of the DEGs. The values > 1 are marked by pink, representing the up-regulated genes of APC^GAL-1−/−^, and the values < − 1 are marked by blue, representing the down-regulated genes of APC^GAL-1−/−^.The values > − 1 and < 1 are marked by grey color. **E** Venn analysis was performed to select the co-expressed DEGs between the APC^GAL-1−/−^ vs. APC^WT^ and APC^GAL-1−/−^ vs. APC^Vector^. *DEGs* differentially expressed genes. **F**, **G** Verification of the transcriptome results on the selected DEGs by qRT-PCR.

The Principal Component Analysis (PCA) were performed (Fig. 4A). The PC2 results showed that APC^GAL-1−/−^ was separated from APC^WT^ and APC^Vector^, and there was few variations between APC^WT^ and APC^Vector^. The differentially expressed gene (DEGs) analysis suggested 173 DEGs existed between APC^WT^ and APC^Vector^ (Fig. 4B), while 1502 DEGs existed between APC^GAL-1−/−^ and APC^WT^ (Fig. 4C) and 1636 DEGs exited between APC^GAL-1−/−^ and APC^Vector^ (Fig. 4D; |log_2_foldchange| > 1, adjusted *P-*value < 0.01). Thus, the analysis results above suggested that APC^Vector^ and APC^WT^ maintained the same gene expression pattern, while APC^GAL-1−/−^ demonstrated distinctive gene expression. Six DEGs between APC^WT^, APC^Vector^, and APC^GAL-1−/−^ (COL18A, TABU1D, BMP2, IGF1, PDGFA, and HERC6) were selected randomly to validate the transcriptome analysis result by RT-PCR (Fig. 4E, F). The results of qRT–PCR and sequencing showed that the expression trends of differential genes were the same, which proved the accuracy of the sequencing.

**Fig. 5:**
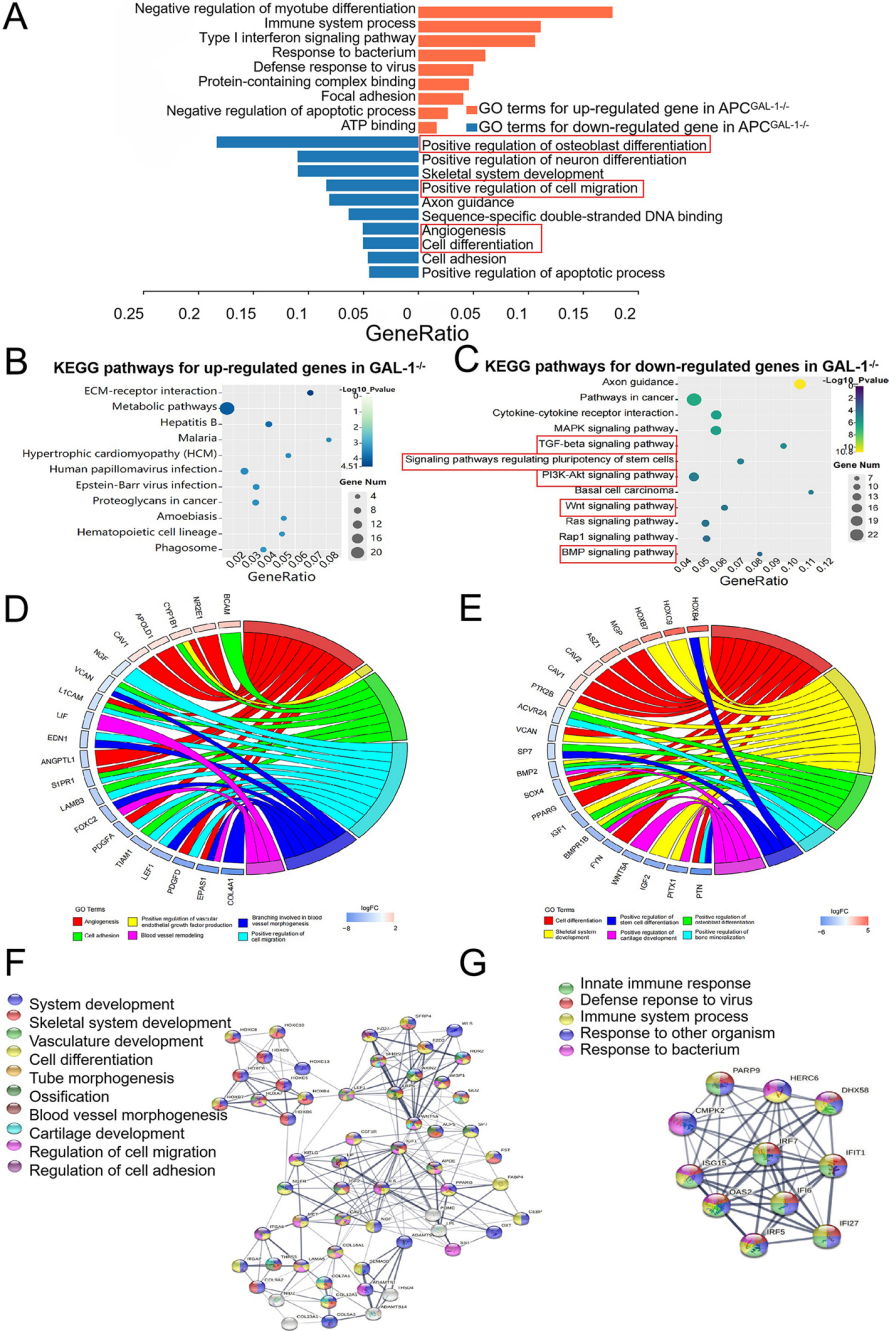
Functional analysis on co-expressed DEGs between the APC^GAL-1−/−^ vs. APC^WT^ and APC^GAL-1−/−^ vs. APC^Vector^. **A** GO analysis in BP was performed on 392 up-regulated (pink bar) and 771 down-regulated (blue bar) DEGs in APC^GAL-1−/−^. Y-axis represents the important GO terms (adjusted *P* < 0.01). **B**, **C** KEGG analysis was performed on the 392 up-regulated (**B**) and 771 down-regulated (**C**) DEGs in APC^GAL-1−/−^, respectively. The horizontal axis represents the gene ratio, the percentage of total DEGs in the given KEGG pathways. The bubble size represents the number of DEGs involved. The bubble color key from white to purple indicates low to high -log10 value of adjusted *P-*values (adjusted *P-*values < 0.01). **D**, **E** Enriched DEGs relating to GO terms on angiogenesis (**D**) and cell differentiation (**E**), respectively. The DEGs interaction network is divided into 17 gene clusters. The two main gene clusters were performed. One is an angiogenesis and bone development-related gene cluster (**F**), and another is an immune response-related gene cluster (**G**)

To further analyze the functions of DEGs of APC^GAL-1−/−^, we generated co-expressed 1163 DEGs from 1502 (APC^GAL-1−/−^ vs. APC^WT^) DEGs and 1636 (APC^GAL-1−/−^ vs. APC^Vector^) DEGs by the Venn analysis (Fig. 4G; |log_2_foldchange| > 1, adjusted *P-*value < 0.01). Most of the co-expressed 1163 DEGS of APC^GAL-1−/−^ were down-regulated (including 771 down-regulated genes and 392 up-regulated genes). The biological process analysis of GO showed that down-regulated genes of APC^GAL-1−/−^ were mainly classified into positive regulation of osteoblast differentiation, positive regulation of cell migration, cell differentiation, and angiogenesis, etc. On the other hand, the up-regulated APC^GAL-1−/−^ genes were mainly classified into immune responses, etc. (Fig. 5A; adjusted *P-*value < 0.01). Given a lot of GO terms relating to angiogenesis and cell differentiation, the co-expressed genes were additionally summarized from angiogenesis-relevant GO terms (Fig. 5D), and the cell differentiation-relevant GO terms (Fig. 5E). The KEGG pathway analysis showed that the down-regulated genes of APC^GAL-1−/−^ were mainly enriched in pathways in PI3K-Akt signaling pathway, TGF-beta signaling pathway and signaling pathways regulating pluripotency of stem cells, etc. (Fig. 5B; adjusted *P-*value < 0.01). The up-regulated genes of APC^GAL-1−/−^ were mainly enriched in pathways in ECM-receptor interaction and hepatitis B, etc. (Fig. 5C; adjusted *P-*value < 0.01). By analyzing the interaction network of 1163 DEGs and clustering them, 17 gene clusters were found, among which there were two main gene clusters. One of the gene clusters was associated with the regulation of angiogenesis and bone development (Fig. 5F), and the other with the regulation of immune responses (Fig. 5G). The above results indicate that GAL-1 regulates the differentiation of APCs, inducing angiogenesis and immune response processes.

## Discussion

To the best of our knowledge, this study is the first to investigate the functions of deer GAL-1 on chondrogenesis and angiogenesis during antler regeneration. Notably, GAL-1 was highly expressed in different developmental stages and related tissues of antler. This phenomenon indicates that GAL-1 may play an irreplaceable role in the whole development and regeneration of antler.

When the male deer (except reindeer, which have antlers for both sexes) reaches puberty, APCs rapidly proliferate and differentiate to form the pedicle and first antler through modified endochondral ossification within a few months [4]. To ensure blood supply of oxygen, energy and nutrients, blood vessels need to elongate at the same rate as the antler tip. Paracrine factors secreted by AP cell-derived progeny promote the elongation of vessels originating from branches of the superficial temporal artery in the subcutaneous vascular layer toward the distal end of the pedicle or antler [32]. The arteries extensively branch in the antler tip and curve into the pre-cartilage layer, cartilage layer, and bone, forming an array of parallel return venous vessels [9, 10]. One of the proteins that may be crucial in the initial vascularization of the antler cartilage is GAL-1. In present study, immunohistochemical results showed that GAL-1 was overexpressed in AP, PP, growth center of antler, and a vascular layer of antler compared with FP. The expression level of GAL-1 protein or mRNA was also significantly higher in ASCs compared to FPCs. This led us to be interested in whether deer GAL-1 regulates the development and angiogenesis of antler. The stimulation of endothelial cells by cell medium and the micro-mass culture of APCs also supported our hypothesis.

It has to be mentioned that angiogenesis is a complex process involving four sequential steps, including (1) degradation of basement membrane by proteases; (2) migration and germination of endothelial cells into interstitial space; (3) endothelial cells proliferation at the migrating tip; (4) formation of the lumen [33]. The formation of intact vessels must rely on autocrine and/or paracrine angiogenic factors to induce migration, proliferation, orientation, extension, and differentiation of endothelial cells, leading to the reconstruction of the basement membrane, lumen formation, and anastomosis with other vessels [34]. Our study used the APCs cell medium to simulate the angiogenesis induced by antler stem cells through exocrine effect using proliferation, migration, and tube formation assays of endothelial cells. The ability of APC^GAL-1−/−^ to induce the vascular formation of endothelial cells was severely impaired, although not totally eliminated. Moreover, adding deer GAL-1 protein to the culture medium also promoted endothelial cell proliferation, migration, and tube formation. These results suggest that APCs induce angiogenesis by secreting GAL-1 with strong angiogenic activity. However, previous studies have shown that GAL-1 overexpression often leads to tumor disordered expansion and an increase in the microvascular area inside the tumor [18, 20]. Inhibition of GAL-1 activity has gradually become a new target for tumor therapy [35]. In contrast, GAL-1 is also overexpressed in antler, but everything is controlled by in a controlled tissue growth unlike cancerous one. The regulatory mechanism is very valuable, which may be unique to antler. In addition, understanding the regulation of GAL-1 mediated angiogenesis in the antler may also help to halt or reduce the uncontrolled equivalent mechanism in some tumours.

Given the wide distribution and high expression of GAL-1 in deer antlers, to further explore its functions and regulatory mechanisms, a comparative transcriptional analysis of APC^WT^, APC^Vector^, and APC^GAL-1−/−^ was performed. We found that down-regulated APC^GAL-1−/−^ genes are mainly related to the upregulation of osteoblast differentiation, positive regulation of cell migration, angiogenesis, and positive regulation of neuron differentiation. Most down-regulated genes promote development and angiogenesis in antlers or other tissues and cells, including Pleiotrophin (PTN), Hypoxia-inducible factor 2 alpha (HIF2), Insulin-like growth factor I (IGF1), and Bone morphogenetic protein 2 (BMP2), etc. Deer GAL-1 may regulate these genes inducing angiogenesis and regulating differentiation of ASCs.

Osteogenesis and angiogenesis are interrelated and tightly regulated processes in growth, repair, and bone remodeling. BMP2 and PTN can promote bone repair by enhancing osteogenesis and new blood vessel formation in vivo or in vitro [36]. PTN is highly expressed in vascular smooth muscle cells in the dermis and the anterior cartilage region of deer antlers, where blood vessels rapidly grow [37]. It may promotes the extension of antler endochondral blood vessels through the chemotactic ability of microvascular endothelial cells and the signal of angiogenesis [38, 39]. IGF-1 is a highly efficient growth regulator, expressed in the mesenchymal layer, pre-cartilage layer, and cartilage layer of deer antlers [40]. IGF-1 promotes the proliferation and differentiation of ASCs and antler chondrocytes by binding with IGF-1R or inhibiting IRS1/2 [41]. HIF2 is a transcription factor that responds to stress during tissue or body hypoxia by regulating neovascularization and anaerobic metabolism [42]. In hyperproliferative tissues that require a large amount of oxygen consumption, such as antlers, Hypoxia is an important biological feature, and HIF hypoxia response elements can directly bind to GAL-1 and promote its expression to induce neovascularization [43]. It should also be noted that down-regulated genes were mainly enriched in PI3K/AKT, TGF-β, and Wnt pathways, which play a crucial role in regulating deer antler development, angiogenesis, skeletal maintenance, and differentiation of ASCs [44, 45].

On the transcriptional level, a noteworthy phenomenon was that up-regulated genes of APC^GAL-1−/−^ were mainly related to immune responses, including the immune system’s responses to bacteria and viruses. According to previous studies, GAL-1 has a powerful immunosuppressive effect by inhibiting T cell proliferation or inducing T cell apoptosis [46–48]. Scar formation in the immune process of wound injury plays a key role in preventing regeneration. How to inhibit the formation of “scar” to stimulate the potential of regeneration has been a hot topic in immune and regenerative medicine research. The wounds are exposed when the antlers fall off, and the blood flows out. In a normal body, immune cells are activated in large numbers, prompting “scarring”. However, the fact is that there was no inflammation after antlers shedding, and the wound with an area of about 10 cm^2^ produced after antler shedding did not leave any scars after healing [49]. Based on previous studies and our transcriptome analysis, a very interesting topic is that GAL-1 may be involved in deer antler regeneration by regulating immune responses.

In summary, we demonstrated that deer GAL-1 promotes angiogenesis and chondrogenesis during stem cell-based regeneration of antler. The present study has not only provided new data support for the mechanism of antler regeneration, but also opened up a new line of research for the investigation of vascularized cartilage. In addition, further exploration of the mechanism may also provide more possibilities for inhibiting tumor growth by controlling the disordered development of blood vessels within the tumor.

## Supplementary Information

**Additional file 1:** **Figure S1.** Tissue sampling sites of AP, PP, FP and antler growth center. A The antlerogenic periosteumis located above the eye sockets on both sides and develops into pedicle during puberty. Note the bulges on both sides. Facial periosteumswas used as a control in this study. B Pedicle periosteumis located below the antler. Pedicle is a permanent bone post derived from AP and does not fall off with antler. Notice the pedicle below the dotted line. C The growth center is about 5 cm on the tip of antler. According to the degree of antler stem cell differentiation, it can be divided into reserve mesenchyme, pre-cartilage, transition zone, and cartilagefrom distal to proximal.

**Additional file 2:** Western-blots images.

**Additional file 3:** **Table S1.** qRT-PCR primers. **Table S2.** CRISPR/gRNAs for GAL-1 GENE. **Table S3.** Summary of transcriptome sequencing data. **Table S4.** Alignment rates of effective RNA-seq sequences on the reference genome.

## Data Availability

The datasets presented in this study can be found in online repositories. The name of the repository and accession number can be found below: NCBI BioSample accession number: PRJNA921710; The link is https://www.ncbi.nlm.nih.gov/biosample.

## Abbreviations

AP: Antlerogenic periosteum
APC^GAL^^-^^1^^−^^/^^−^: APCs with the GAL-1 gene knocked out
APCs: Antlerogenic periosteal cells
APC^vector^: APCs infected with empty vector
APC^WT^: Wild-type APCs
ASCs: Antler stem cells
C: Cartilage
DEGs: Differentially expressed genes
FP: Facial periosteum
FPCs: Facial periosteal cells
GAL-1: Galectin-1
HUVECs: Human umbilical vein endothelial cells
PC: Pre-cartilage
PP: Pedicle periosteum
PPCs: Pedicle periosteal cells
RM: Reserve mesenchyme
RMCs: Reserve mesenchymal cells
TZ: Transition zone
PTN: Pleiotrophin
HIF2: Hypoxia-inducible factor 2 alpha
IGF1: Insulin-like growth factor1
BMP2: Bone morphogenetic protein 2
