# Supplementary material for: Galectin-1 promotes angiogenesis and chondrogenesis during antler regeneration
Source: Cell Mol Biol Lett. 2023 May 15;28:40. doi: 10.1186/s11658-023-00456-7 (PMC10184426; doi:10.1186/s11658-023-00456-7)
Supplement: Supplementary file 1 — Additional file 1: Figure S1. Tissue sampling sites of AP, PP, FP and antler growth center. A The antlerogenic periosteumis located above the eye sockets on both sides and develops into pedicle during puberty. Note the bulges on both sides. Facial periosteumswas used as a control in this study. B Pedicle periosteumis located below the antler. Pedicle is a permanent bone post derived from AP and does not fall off with antler. Notice the pedicle below the dotted line. C The growth center is about 5 cm on the tip of antler. According to the degree of antler stem cell differentiation, it can be divided into reserve mesenchyme, pre-cartilage, transition zone, and cartilagefrom distal to proximal. [file 11658_2023_456_MOESM1_ESM.pdf]

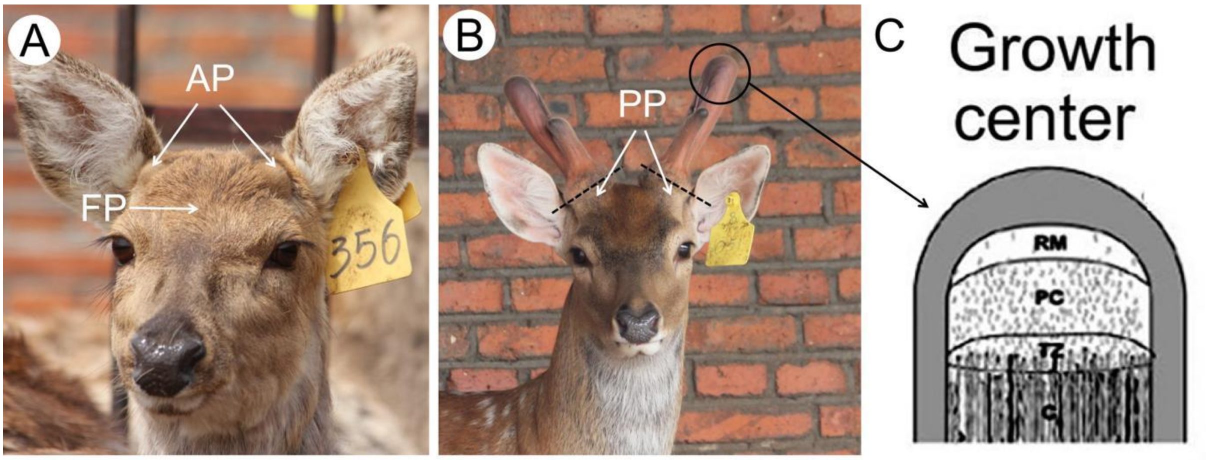

Additional file 1: Figure. S1. Tissue sampling sites of AP, PP, FP and antler growth center. **(A)** The antlerogenic periosteum (AP) is located above the eye sockets on both sides and develops into pedicle during puberty. Note the bulges on both sides. Facial periosteums (FP) was used as a control in this study. **(B)** Pedicle periosteum (PP) is located below the antler. Pedicle is a permanent bone post derived from AP and does not fall off with antler. Notice the pedicle below the dotted line. **(C)** The growth center is about 5cm on the tip of antler. According to the degree of antler stem cell differentiation, it can be divided into reserve mesenchyme (RM), pre-cartilage (PC), transition zone (TZ), and cartilage (C) from distal to proximal.
