# Supplementary material for: Galectin-1 promotes angiogenesis and chondrogenesis during antler regeneration
Source: Cell Mol Biol Lett. 2023 May 15;28:40. doi: 10.1186/s11658-023-00456-7 (PMC10184426; doi:10.1186/s11658-023-00456-7)
Supplement: Supplementary file 2 — Additional file 2: Western-blots images. [file 11658_2023_456_MOESM2_ESM.pdf]

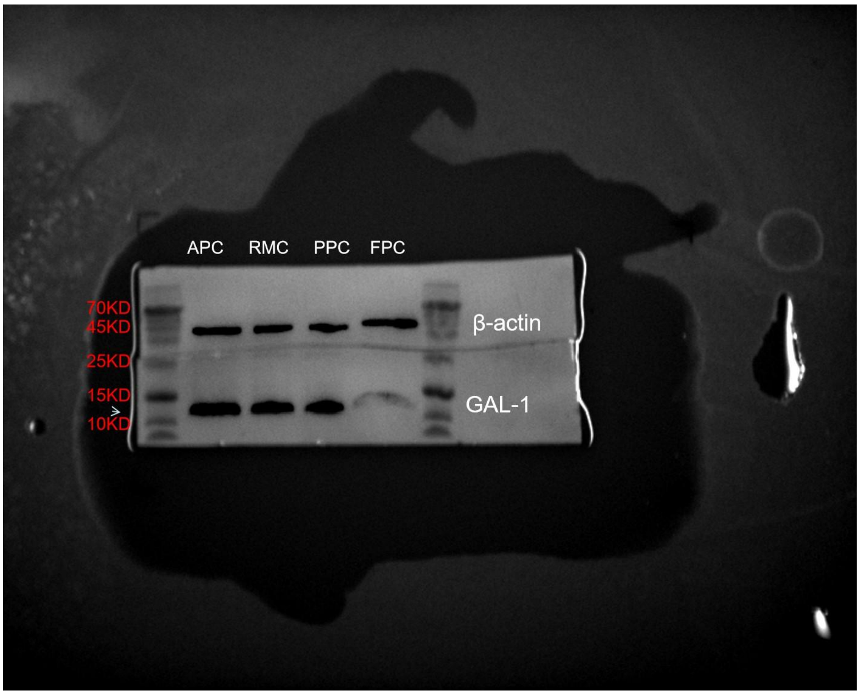

Western-blots for determination of GAL-1 protein expression (1)

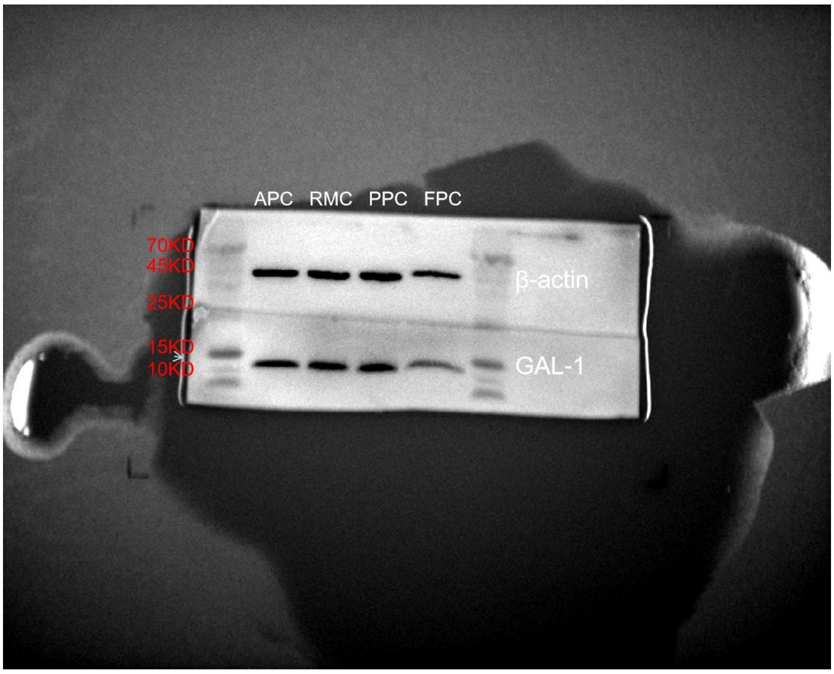

Western-blots for determination of GAL-1 protein expression (2)

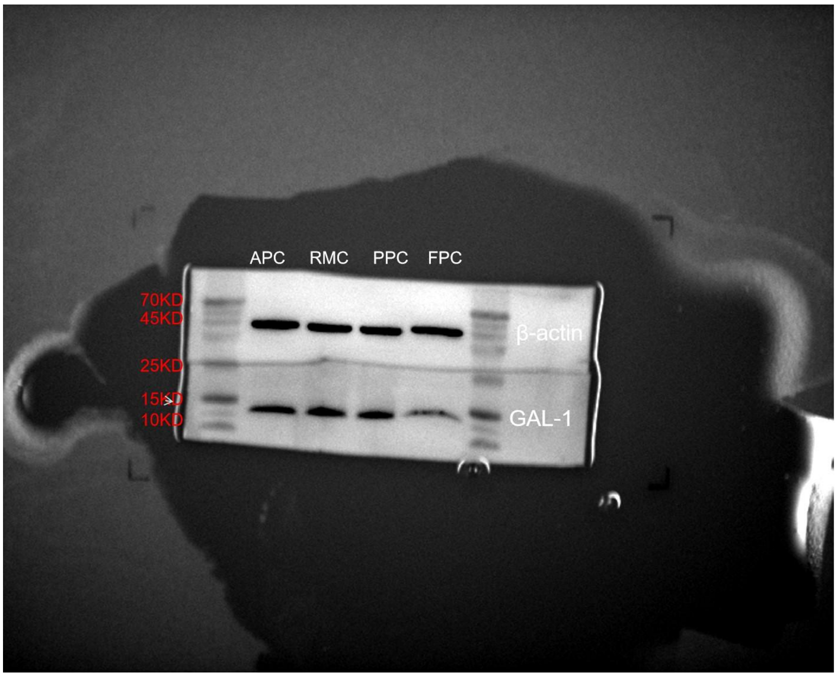

Western-blots for determination of GAL-1 protein expression (3)

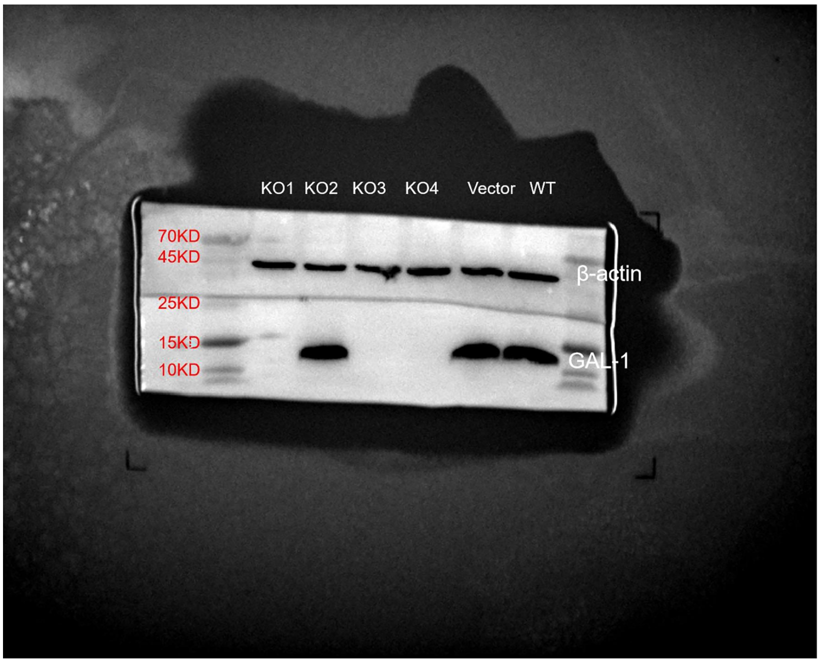

Identify the GAL-1 knockout effect of antlerogenic periosteal cells (APC) by Western-blot
